# Neoadjuvant Targeted Therapy in Non–Small Cell Lung Cancer and Its Impact on Surgical Outcomes

**DOI:** 10.1016/j.atssr.2022.11.014

**Published:** 2022-11-25

**Authors:** Mark Sorin, Caroline Huynh, Merav Rokah, Laurie-Rose Dubé, Roni Rayes, Linda Ofiara, Benjamin Shieh, Scott Owen, Pierre Olivier Fiset, Sophie Cammilleri-Broët, Sara Najmeh, Jonathan Cools-Lartigue, Lorenzo Ferri, Jonathan D. Spicer

**Affiliations:** 1 Rosalind and Morris Goodman Cancer Research Centre, McGill University, Montréal, Quebec, Canada; 2 Department of Human Genetics, McGill University Health Centre, Montréal, Quebec, Canada; 3 Department of Surgery, McGill University, Montréal, Quebec, Canada; 4 Faculty of Medicine, McGill University, Montréal, Quebec, Canada; 5 Department of Oncology, McGill University Health Centre, Montréal, Quebec, Canada; 6 Department of Pathology, McGill University, Montréal, Quebec, Canada

## Abstract

**Background:**

The evidence for neoadjuvant targeted therapy in non–small cell lung cancer is limited, with 2 phase 3 trials currently recruiting and no approved indications.

**Methods:**

We describe our experience with the use of neoadjuvant targeted therapy for patients with operable non–small cell lung cancer.

**Results:**

Our focus is on surgical outcomes, which represent an underreported aspect of the patient trajectory. We argue that surgical outcomes are an essential feature of this strategy with significant potential benefits and risks.

**Conclusions:**

Overall, the patient experience can be significantly affected by the use of neoadjuvant targeted therapy and its impact on surgical planning, strategy, and outcomes.

**In Short**

▪ There is currently limited evidence for the use of neoadjuvant tyrosine kinase inhibitors (TKIs) in non–small cell lung cancer.
▪ We sought to describe our experience with 5 patients with non–small cell lung cancer treated by neoadjuvant TKIs.
▪ We highlight the surgical outcomes of these patients and demonstrate why the reporting of surgical outcomes in future neoadjuvant TKI phase 3 trials is essential.

Patients with operable non–small cell lung cancer (NSCLC) are a growing fraction of newly diagnosed patients thanks to early detection and screening programs. Nearly 50% of these patients will have actionable mutations for which there are approved drugs in the metastatic setting.^1^ In recent years, neoadjuvant therapy has gained a lot of attention. Neoadjuvant therapy allows downstaging of unresectable tumors, rendering them resectable and simultaneously eliminating micrometastatic disease.^2^ The long-term benefits of investigational agents in the neoadjuvant setting remain unclear for some therapeutic drug classes in NSCLC. Initial results from CheckMate 816 show that the addition of immunotherapy to chemotherapy in the neoadjuvant setting led to a 24% rate of pathologic complete response compared with a 2% rate for neoadjuvant chemotherapy alone without imparting a negative impact on adverse event rates or surgical resection rates.^3^ That said, one of the most striking findings from the results available from CheckMate 816 relates to the exploratory surgical outcomes. Overall, patients treated with neoadjuvant chemotherapy-nivolumab had shorter operations, and fewer required open surgical procedures, conversion to open surgery, or pneumonectomy to resect remaining disease. These are all important end points for the patient experience, and no perioperative therapy has ever shown such impact on the conduct of the surgical resection.

For targeted therapy, the results of the ADAURA trial have brought tyrosine kinase inhibitors (TKIs) to the resectable stages of EGFR-mutated lung cancer with a dramatic improvement in disease-free survival.^4^ The evidence for the use of neoadjuvant EGFR TKIs is limited, as was concluded by 2 independently performed meta-analyses.^5^^,^^6^ Future phase 3 randomized trials such as the NeoADAURA trial, which will evaluate neoadjuvant osimertinib with or without chemotherapy vs chemotherapy alone before surgery, will provide evidence for the use of neoadjuvant TKIs.^7^ Given the limited evidence for this approach so far, we sought to describe our experience with neoadjuvant TKI-treated patients, with a focus on surgical outcomes.

## Patients and Methods

Patients were selected from a prospectively collected institutional NSCLC database of patients seen as part of the thoracic surgery service at the McGill University Health Centre from January 2015 to July 2022. Five patients met our search criteria of having received neoadjuvant TKI treatment and were included as part of this retrospective analysis. Demographic characteristics (sex, age), baseline characteristics (smoking status), preoperative or diagnostic data (tests and procedures, neoadjuvant therapy, clinical staging), pathological data (pathological staging, lymph node status, margin status, histological type), and operative data (approach) were collected. Lymph node station sampled can be found in Table 1. All available computed tomography (CT) scans at diagnosis and after completion of neoadjuvant therapy but before surgery were studied. For each CT slice, the outline of the tumor was drawn using a pencil selection tool, permitting a higher level of precision. Tumor volumes were then automatically calculated for each tumor using the picture archiving and communication system imaging software that sums the area of tumor for each CT slice. Research ethics approval was obtained from the Research Board of McGill University (#2022-8070).

**Table 1:** Lymph Node Stations for Each Patient

Patient	Lymph Node Station
75-year-old woman	5, 7, 10L, 11L
62-year-old woman	4R, 7, 11R
68-year-old woman	2R, 4R, 7, 9R, 10R, 11R
29-year-old woman	2R, 4R, 7, 10R, 11R, 12R
74-year-old woman	2R, 3R, 4R, 7, 10R, 11R

## Results

We retrospectively reviewed 5 cases of neoadjuvant TKI-treated NSCLC patients from January 2015 to July 2022 at a single center. In terms of treatment trajectories (Figure), patient 1 was a 75-year-old female ex-smoker with clinical stage IIIa (cT4N0M0; T4 due to tumor size) EGFR-mutated invasive acinar-predominant lung adenocarcinoma. She was treated with neoadjuvant gefitinib; the tumor was then downstaged to pathologic stage Ib (ypT2aN0) after a left lower video-assisted thoracoscopic surgery (VATS) lobectomy and mediastinal node dissection with microscopically positive margins. Despite a microscopically positive parenchymal margin at final pathologic examination, the patient is without thoracic recurrence now >2 years after surgery and without adjuvant radiation therapy. Her tumor decreased in size from 10 × 5.7 cm (348.48 cm^3^) to 4.3 × 3.5 cm (63.76 cm^3^) after neoadjuvant TKI treatment, an 82% reduction in tumor volume. She had a 2-day length of stay after surgery. The patient went on to receive 4 cycles of adjuvant cisplatin and pemetrexed. Five months later, the patient presented with a brain recurrence causing dysphasia and cognitive changes. She underwent brain metastasectomy through craniotomy followed by osimertinib. She remains alive without disease 26 months after her initial diagnosis.

**Figure:**
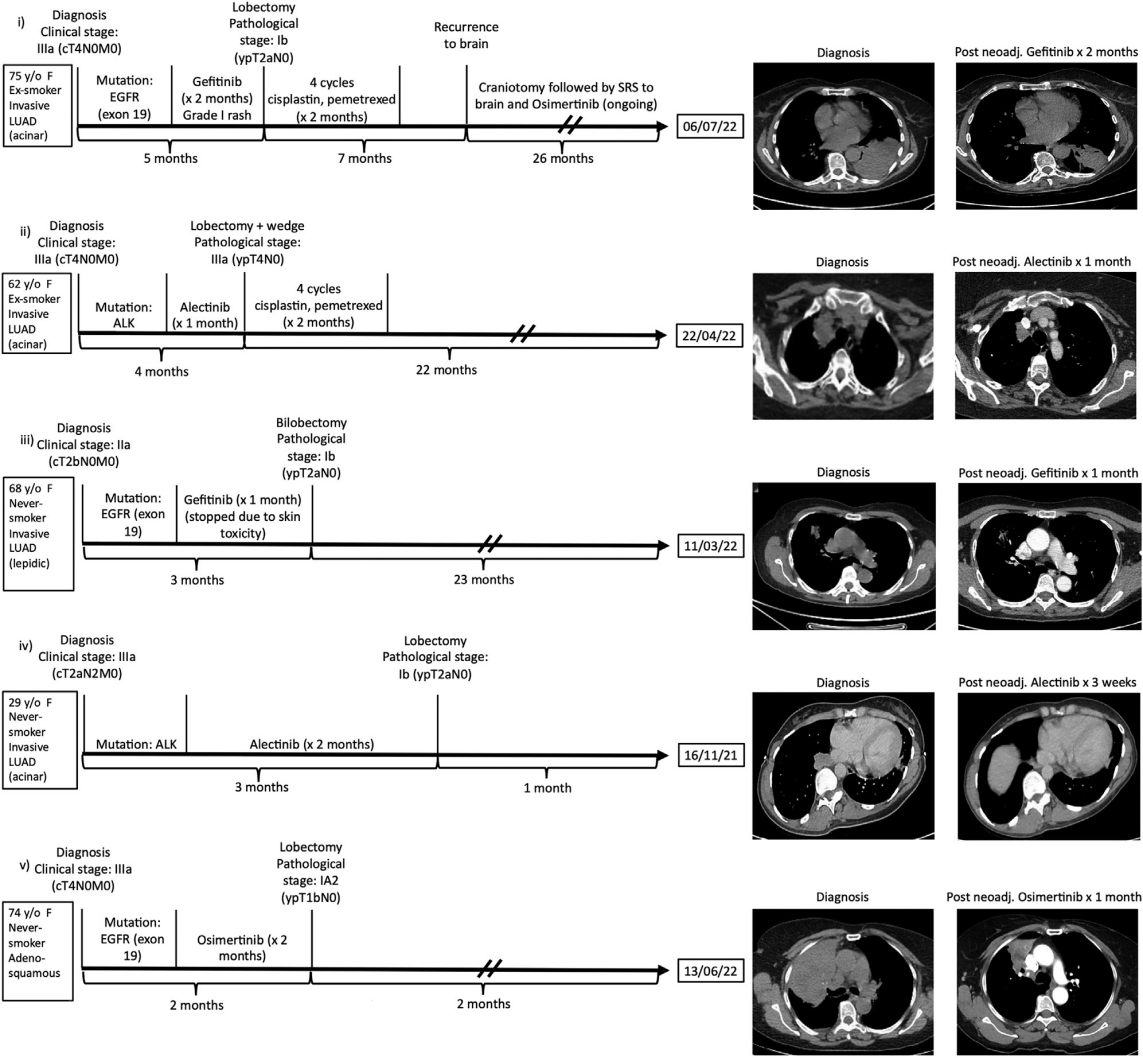
Treatment trajectories of neoadjuvant tyrosine kinase inhibitor (TKI)–treated patients with non–small cell lung cancer. Timelines include mutation status, clinical and pathologic stage, type of neoadjuvant TKI used, type of surgery used, type of adjuvant therapy used, and information on recurrence. Computed tomography scans at diagnosis and after neoadjuvant (Post neoadj.) TKI treatment are also provided. (F, female; LUAD, lung adenocarcinoma; SRS, stereotactic radiosurgery; y/o, year old.)

Patient 2 was a 62-year-old female ex-smoker with clinical stage IIIa (cT4N0M0; T4 due to invasion of the mediastinum and superior vena cava) ALK-mutated invasive acinar-predominant lung adenocarcinoma. The mass was invading the mediastinal fat and may have been impinging on the superior vena cava. After 1 month of alectinib, her tumor decreased in size from 3.8 × 3.0 cm (31.91 cm^3^) to 2.8 × 1.9 cm (11.21 cm^3^), a 65% reduction in tumor volume. After a right upper VATS lobectomy and wedge resection and mediastinal node dissection (R0), she was discharged on the second postoperative day. Final pathologic examination demonstrated visceral pleural invasion and structural invasion of the mediastinum with negative margins, resulting in stage IIIa (ypT4N0) disease. She went on to receive adjuvant cisplatin and pemetrexed. She remains without evidence of disease now 22 months after surgery.

Patient 3, a 68-year-old female never-smoker, had EGFR-mutated clinical stage IIa (cT2bN0M0) lepidic-predominant adenocarcinoma, for which she received gefitinib that was stopped after a month because of skin toxicity. This led to a slight interval decrease in tumor volume from 3 cm^3^ to 1.82 cm^3^, a 39% reduction. She then underwent a right upper VATS bilobectomy and mediastinal node dissection (R0). She was discharged on the first postoperative day. Final pathologic examination demonstrated lymphovascular invasion, lepidic predominant, stage Ib (ypT2aN0). She is now 23 months after surgery with no evidence of disease*.*

Patient 4 was a 29-year-old woman with an ALK-mutated stage IIIa (cT2aN2M0) acinar-predominant adenocarcinoma. She had multistation N2 disease (levels 2R, 4R, and 7) and had been offered concurrent chemoradiation at an outside institution. She declined chemoradiation in favor of neoadjuvant alectinib followed by surgery. She received 2 months of neoadjuvant alectinib with a decrease in tumor size from 1.8 cm^3^ to 0.43 cm^3^, a 76% reduction. She underwent a right VATS lower lobectomy with lymph node dissection and was discharged on the third postoperative day. On final pathologic examination, she was found to have lymphovascular and visceral pleural invasion, and the tumor was downstaged to stage Ib (ypT2aN0) after an R0 resection with a major pathologic response (<10% remaining viable tumor cells). She resumed alectinib on postoperative day 3 and is scheduled to undergo adjuvant chemotherapy.

Patient 5 was a 74-year-old female never-smoker with an exon 19 EGFR-mutated stage IIIa (cT4N0M0; T4 due to tumor size) adenosquamous carcinoma. She received neoadjuvant osimertinib for 2 months, which led to a decrease in tumor volume from 378.23 cm^3^ to 37.63 cm^3^, a 90% reduction. She underwent a right VATS upper lobectomy with lymph node dissection. The tumor was downstaged to stage Ia2 (ypT1bN0) with a major pathologic response, and she is scheduled to receive adjuvant osimertinib.

## Comment

In our case series, 2 of 5 patients achieved a major pathologic response, no patients achieved complete pathologic response, and no patients experienced surgical complications. All patients were operated on by a minimally invasive technique, with none requiring conversion to open surgical procedures. Postoperative length of stay ranged from 1 to 3 days. Overall, 4 of 5 patients had downstaging after neoadjuvant TKI treatment with direct impact on the surgical approach (Table 2). The anecdotal nature of these findings is an obvious limitation, and it is impossible to say whether the surgical and clinical outcomes of these patients would have been worse or different had they not received neoadjuvant TKI therapy. However, in our judgment, the use of neoadjuvant TKI simplified the surgical experience for these patients. For example, patient 1 had a large tumor that would have required a large incision simply to remove the mass. However, with significant response, a relatively simple VATS lower lobectomy was performed, allowing the patient to be discharged on the second postoperative day. For patient 2, the mass was invading the mediastinal fat and may have been impinging on the superior vena cava. After 1 month of alectinib, there was significant regression, allowing an uncomplicated VATS resection, and again, she was discharged on the second postoperative day. In patient 4, neoadjuvant therapy offered her a radiation-sparing approach to her local control, which may be important in a 29-year-old patient. She also had downstaging from multistation N2 to N0, which is encouraging with respect to local control of her disease. Patient 5 would have undergone a thoracotomy with bronchial sleeve before neoadjuvant treatment but instead underwent a VATS lobectomy after 2 months of osimertinib. An example of the surgical approach after neoadjuvant TKI therapy can be observed in the Video. Overall, neoadjuvant therapy has the potential to allow less morbid local therapy compared with adjuvant therapy. Future phase 3 trials investigating the effects of neoadjuvant TKIs should offer detailed and systematic reporting of the surgical outcomes in addition to survival, response, and pathological end points.

**Table 2:** Proposed Surgical Approach at Diagnosis Before Neoadjuvant TKI Treatment and Surgery Performed After Neoadjuvant TKI Therapy

Patient	Surgical Approach Before Neoadjuvant Therapy	Surgical Approach After Neoadjuvant Therapy
75-year-old woman	Thoracotomy LUL lobectomy	VATS LUL lobectomy
62-year-old woman	Thoracotomy RUL lobectomy, SVC resection and reconstruction	VATS RUL lobectomy
68-year-old woman	VATS upper bilobectomy	VATS upper bilobectomy
29-year-old woman	Thoracotomy RLL	VATS RLL lobectomy
74-year-old woman	Thoracotomy RUL sleeve lobectomy	VATS RUL lobectomy, resection of azygos vein

LUL, left upper lobe; RLL, right lower lobe; RUL, right upper lobe; SVC, superior vena cava; VATS, video-assisted thoracoscopic surgery.

In particular, it is crucial for such studies to comment on expected surgical approach at presentation and to compare this with the operation carried out after treatment in a prospective controlled fashion. As an example, the increased morbidity linked to extended lung resections such as pneumonectomy is well described in comparison to lobectomy or segmentectomy. Indeed, this is important as pneumonectomy is associated with higher 30-day mortality than lobectomy and worse quality of life after surgery, including worse physical functioning, role functioning, social functioning, and general pain.^8^^,^^9^ As an example, the CheckMate 816 study found a 43% reduction in patients with stage IIIa disease who required pneumonectomies in the chemotherapy-nivolumab patients vs chemotherapy alone (17% vs 30%) and a 45% reduction in conversion from minimally invasive to open surgical procedures (11% vs 20%, respectively) with the addition of nivolumab.^3^ Conversion from minimally invasive to open surgeries has also been associated with higher 30-day mortality, longer length of stay, and increased blood loss compared with minimally invasive surgery alone.^9^ As such, interventions that enable lesser lung resections have the potential to dramatically improve the patient experience of curative therapy.

Our anecdotal experience with neoadjuvant targeted therapy reveals certain aspects of the care trajectory that can be optimized by such an approach. Most data on neoadjuvant targeted therapy involve EGFR mutant patients, with a growing list of approved agents for various other mutations to be tested in the coming years. Future and ongoing neoadjuvant targeted therapy trials should report detailed surgical outcome data. The patient experience is significantly affected by the influence of neoadjuvant treatment on surgical decision-making. These effects have pivotal consequences on resulting quality of life, incurred morbidity of treatment, and survival.

## Supplementary Data

Lobectomy post TKI
